# Hints for Gentle Submacular Injection in Non-Human Primates Based on Intraoperative OCT Guidance

**DOI:** 10.1167/tvst.10.1.10

**Published:** 2021-01-07

**Authors:** Gavin S. W. Tan, Zengping Liu, Tanja Ilmarinen, Veluchamy A. Barathi, Caroline K. Chee, Gopal Lingam, Xinyi Su, Boris V. Stanzel

**Affiliations:** 1 Singapore National Eye Centre, Singapore, Singapore; 2 Singapore Eye Research Institute, Singapore, Singapore; 3 Department of Ophthalmology, Yong Loo Lin School of Medicine, National University of Singapore, Singapore, Singapore; 4 Institute of Molecular and Cell Biology, A*STAR (Agency for Science, Technology and Research), Singapore, Singapore; 5 Faculty of Medicine and Health Technology, Tampere University, Tampere, Finland; 6 Ophthalmology Academic Clinical Research Program, DUKE-NUS Medical School, Singapore, Singapore; 7 Department of Ophthalmology, National University Hospital, Singapore, Singapore; 8 Eye Clinic Sulzbach, Knappschaft Hospital Saar, Sulzbach, Saar, Germany

**Keywords:** submacular surgery, fovea, non-human primates, intraoperative OCT, Advanced Therapy Medicinal Products

## Abstract

**Purpose:**

Delivery of Advanced Therapy Medicinal Products to the submacular space is increasingly evolving into a therapeutic modality. Cell replacement for age-related macular degeneration (AMD) and gene therapy for RPE65 are recent successful examples. Herein, a nonhuman primate (NHP) model was used to investigate surgical means to detach the macula.

**Methods:**

Sixteen eyes of 13 healthy macaques underwent a 25-gauge vitrectomy and subretinal injection of balanced salt solution monitored by microscope-integrated intraoperative optical coherence tomography (miOCT). The animals were followed with OCT and histology.

**Results:**

The miOCT monitoring allowed a more precise definition of surgical trauma ranging from an initial full-thickness foveal tear, or induction of a cystoid macular edema (CME), until no foveal defect was discernible, as the technique improved. However, as the subretinal fluid wave detached the fovea, the aforementioned lesions formed, whereas persistent retinal adhesion reproducibly proved to remain in the distal parafoveal semi-annulus. Measures to reduce foveal trauma during submacular fluid injection included reducing intraocular pressure, injection volume, and velocity, as well as the retinal location for bleb initiation, use of a vitreous tamponade, and a dual-bore subretinal cannula.

**Conclusions:**

A stable very low intraocular pressure and careful subretinal injection may avoid tangential macular stretching or mechanical CME formation, while vitreous tamponade may facilitate a more lamellar subretinal flow, all thereby reducing foveal trauma during submacular injection in NHP.

**Translational Relevance:**

These results can be relevant to any submacular surgery procedure used today, as they synergistically reduce the risk of compromising foveal integrity.

## Introduction

Submacular surgery was developed in the 1990s to remove choroidal neovascularization (CNV) from the subretinal space.^1^^–^^3^ Subsequently, the Submacular Surgery Trials research group did not find a benefit for CNV removal^4^^,^^5^ and combined with the advent of antivascular endothelial growth factor injections thereafter,^6^ there was a decline in submacular surgery for age-related macular degeneration (AMD).^7^ Recently however, submacular surgery has re-emerged as a rapidly evolving therapeutic modality. In patients with thick submacular hemorrhage, subretinal injection of tissue plasminogen activator and pneumatic displacement has been shown to improve vision in CNV and polypoidal choroidal vasculopathy.^8^^,^^9^ The development of current gene therapy for inherited retinal diseases in clinical trials, including RPE65-mediated inherited retinal dystrophy and choroideremia,^10^^–^^13^ requires the delivery of the viral vector safely into the subretinal space. Retinal pigment epithelial (RPE) cell replacement therapy derived from pluripotent stem cells will also require the delivery of cell suspension or sheets into the submacular space.^14^^–^^17^ Parafoveal adhesions, which have been suggested to prevent the closure of large persistent macular holes,^18^ can inhibit successful detachment of the fovea and delivery of a therapeutic product into the subretinal space.

There are limited data available in the literature that address surgical techniques to ensure atraumatic handling of the macula.^19^ The fovea, however, is the weakest mechanical point during submacular maneuvers and its surgical vulnerability is described in several gene therapy studies.^13^^,^^20^^,^^21^ The availability of integrated intraoperative optical coherence tomography (miOCT) provides surgeons with the ability to dynamically evaluate in 4D—in space and time—the microstructural changes in the macula during a subretinal procedure.^22^^,^^23^ In this study, we describe dynamic changes in the macula of nonhuman primates (NHP) in response to a subfoveally-induced retinal detachment using both intraoperative and postoperative OCT. From prior literature, we surmised that factors such as intraocular pressure,^24^ use of tamponade,^12^ type of injection syringe and cannula,^25^^,^^26^ and control of injection by manual or machine-actuated pressure could affect the outcome of the procedure.^11^^,^^21^^,^^27^ Our goal was to obtain immediate feedback on our surgical technique using the miOCT to inform modification of surgical technique and parameters to optimize detachment and minimize structural damage in NHP.

## Methods

### Animals

A total of 13 cynomolgus macaques aged four to six years and weighing 3.0 to 5.0 kg were purchased from SingHealth Experimental Medicine Center, Singapore. All procedures were reviewed and approved by the SingHealth Institutional Animal Care and Use Committee (IACUC, Singapore; AAALAC accredited). Bilateral surgery approval (unilateral eye surgery at a time and given a two-week recovery period to operate on the second eye) was given in this approved IACUC study protocol. All animals were handled in accordance with the Association for Research in Vision and Ophthalmology (ARVO) Statement for the Use of Animals in Ophthalmic and Vision Research.

### Animal Surgery

Detailed surgical procedures in NHP for subretinal delivery of retinal cell therapeutics were previously described.^28^ In brief, animals received induction of anesthesia by atropine (0.05 mg/kg) and ketamine (10 mg/kg), and general anesthesia was induced with 3% isofluorane and maintained with 1% to 2% isofluorane. Pupils were dilated with tropicamide 1% (Mydriacyl; Alcon, Geneva, Switzerland) and phenylephrine hydrochloride 2.5% (Mydfrin; Alcon) eye drops before the surgery was started.

A 25-gauge (G) four-port vitrectomy (infusion, hand-held or chandelier endoillumination, and two working ports) was performed with either an Alcon Constellation or Bausch & Lomb Stellaris PC machine (Bausch & Lomb, Rochester, NY, USA) with general anesthesia. A noncontact wide-angled 128° fundus lens (Resight; C. Zeiss Meditec, Jena, Germany) was used, which was attached to a surgical microscope equipped with an miOCT (OPMI-Lumera 700 with integrated intraoperative OCT, C. Zeiss Meditec). After core vitrectomy, triamcinolone-assisted posterior vitreous detachment (PVD) was performed, and the vitreous skirt was removed up to the vitreous base.

**Figure 1.**
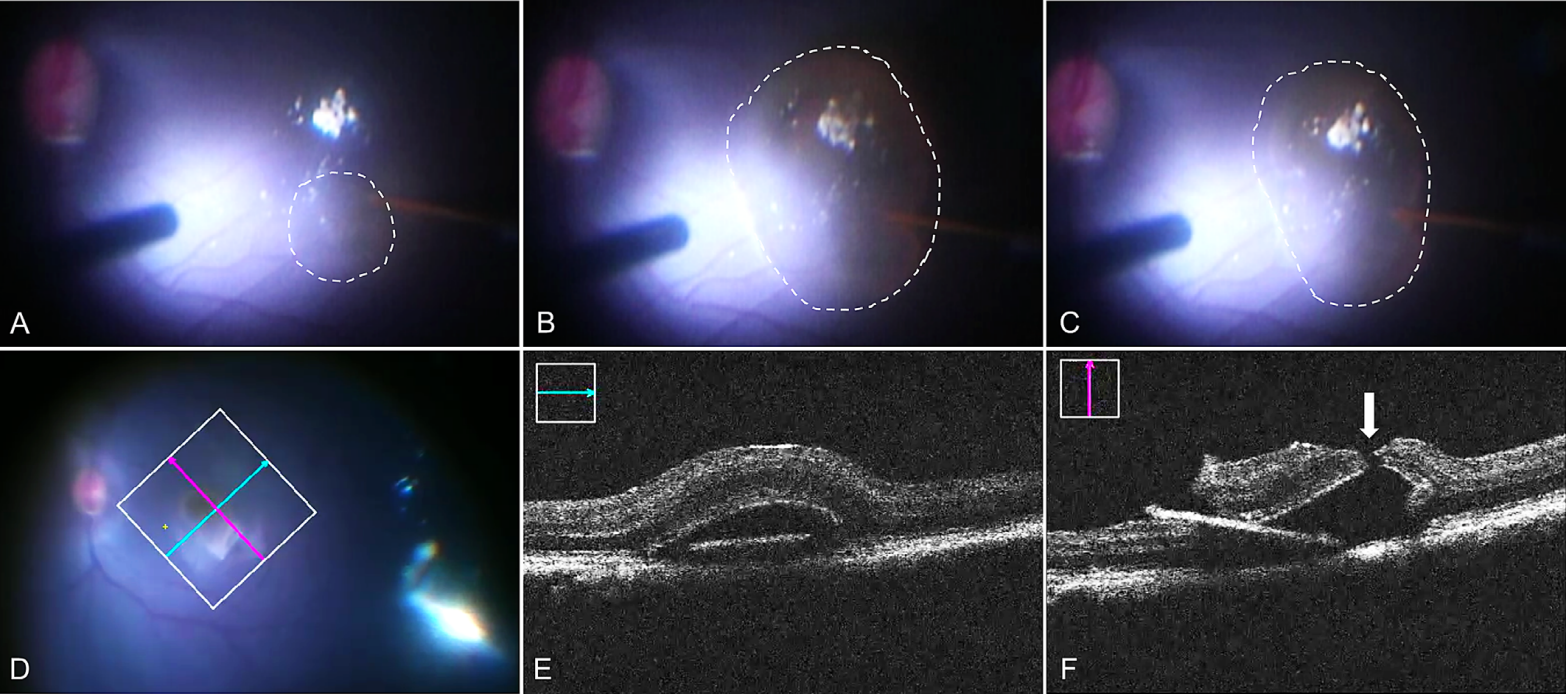
Foveal detachment raised under Condition 1. (A) Retinal bleb was initiated by a 38G cannula (*white circle*) at the superior temporal side. (B) Maximum bleb (*white circle*) reached by one single injection. (C) Bleb size (*white circle*) became smaller when the cannula was still inside the bleb. (D–F) Intraoperative OCT images. *Green line* and *pink line* in D indicate the OCT images in E and F, respectively. *White arrow* in F points to the fovea tear.

**Figure 2.**
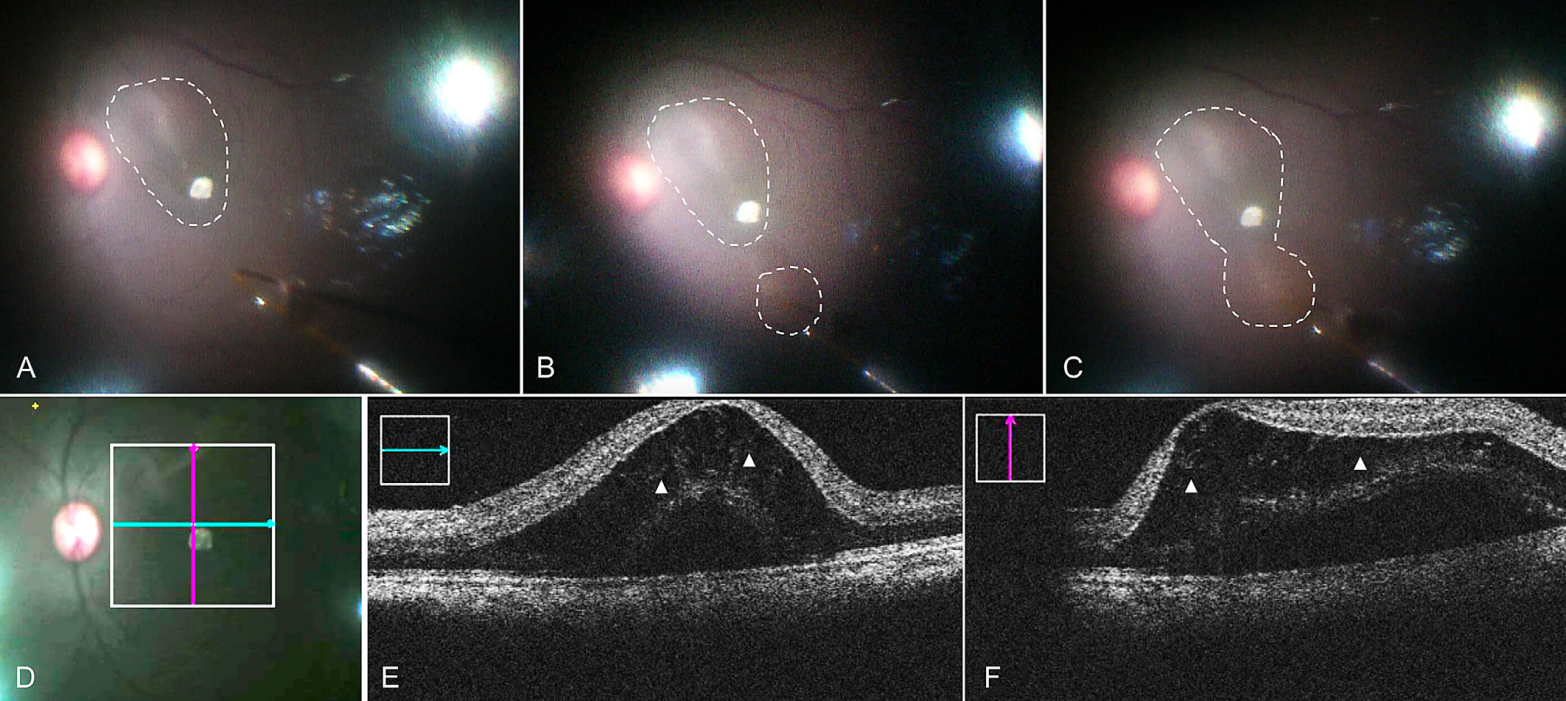
Foveal detachment raised under Condition 2. (A) The first retinal bleb was raised at inferior nasal side (*white circle*), which was not fully detached the fovea. (B) The second bleb was initiated at superior temporal side (*white circle*). (C) Two blebs merged into one (*white circle*) and detached the fovea. (D–F) Intraoperative OCT images. *Green line* and *pink line* in D indicated the OCT images in E and F, respectively. *White triangles* in E and F indicate the severe intraretinal edema.

**Figure 3.**
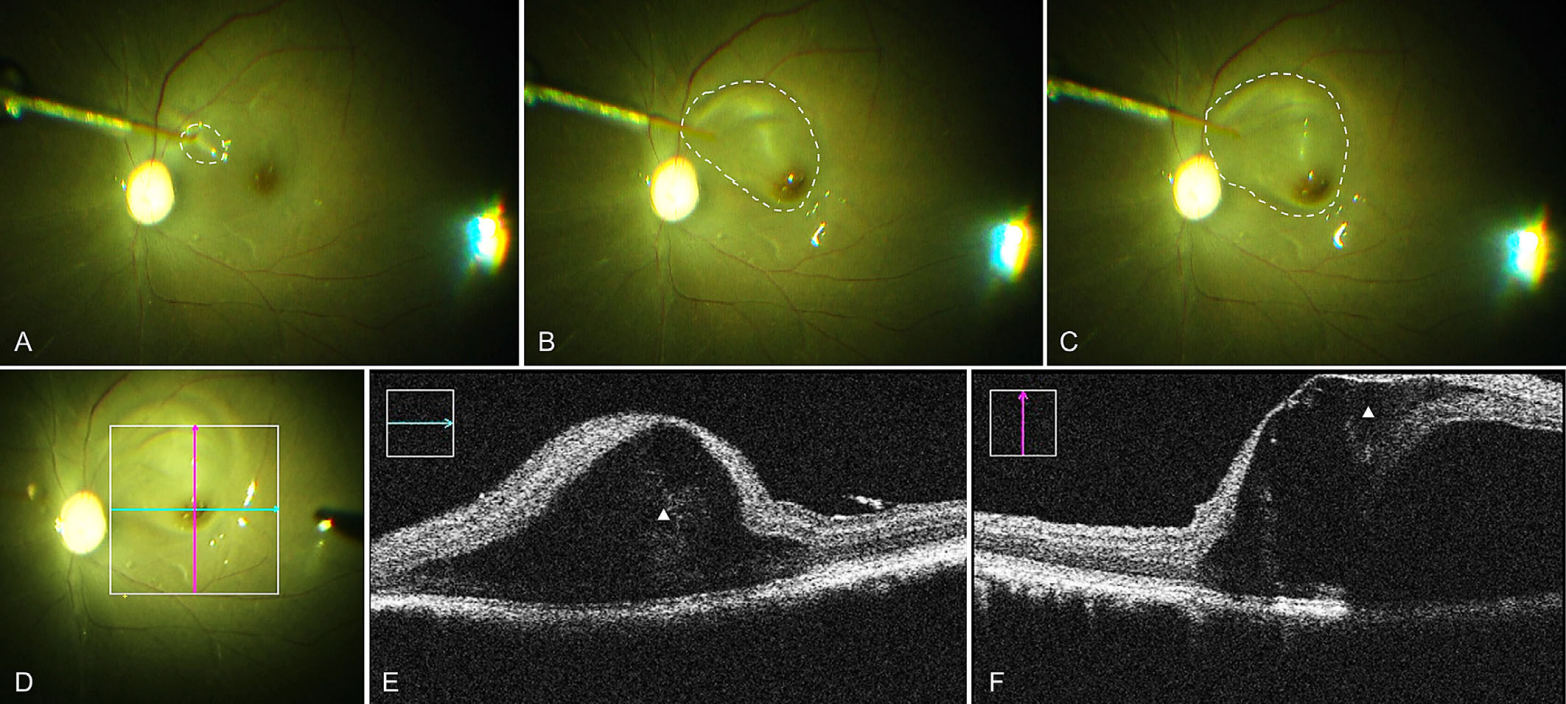
Foveal detachment raised under Condition 3. (A) The retinal bleb was initiated at inferior nasal side (*white circle*). (B) The bleb reached to fovea region and detached one side of the fovea (*white circle*). (C) The bleb did not go further to detach the other side of the fovea and increase the bleb size in vertical direction (*white circle*). (D–F) Intraoperative OCT images. *Green line* and *pink line* in D indicated the OCT images in E and F, respectively. *White triangles* in E and F indicate the severe intraretinal edema.

**Figure 4.**
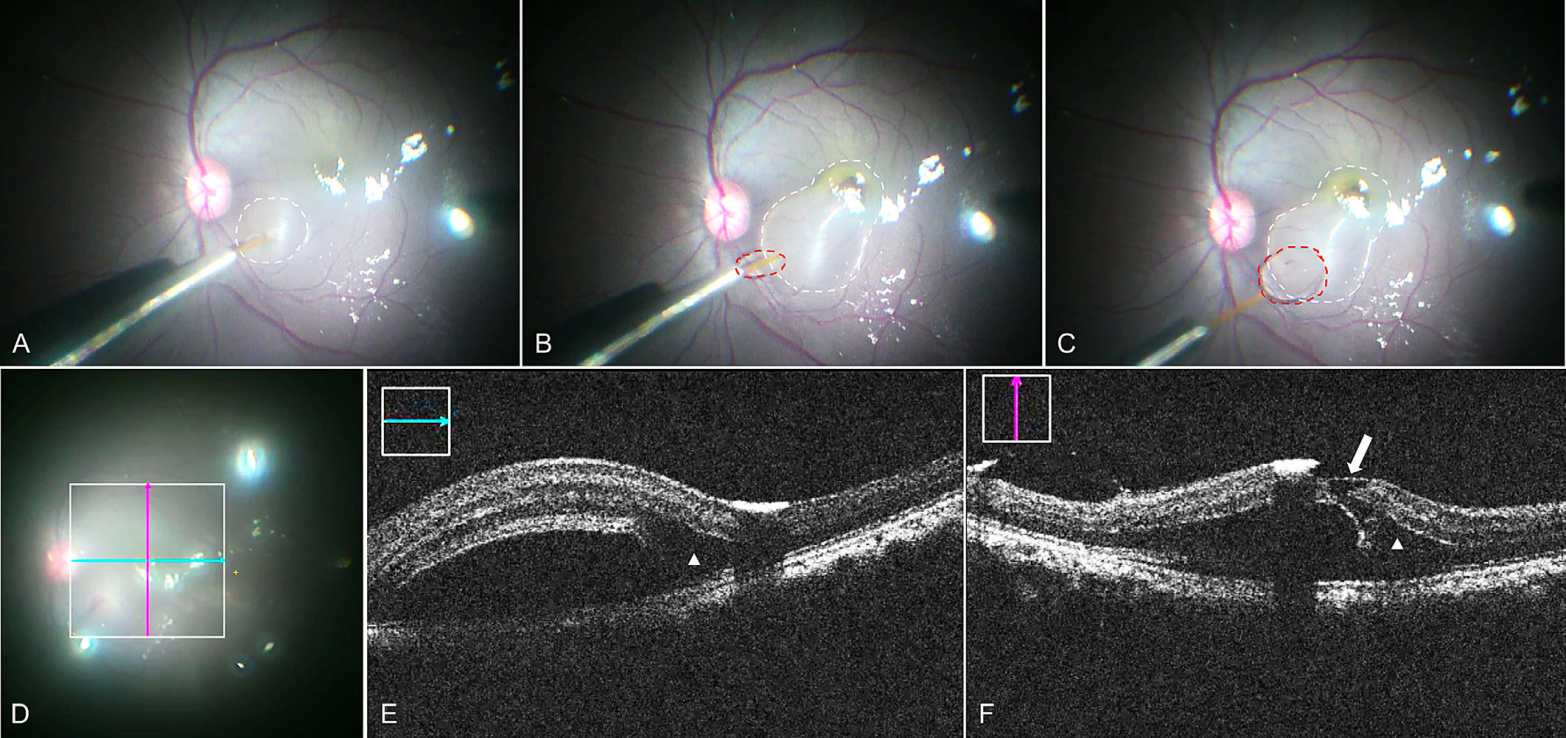
Foveal detachment raised under Condition 4. (A) The retinal bleb was initiated at superior nasal side (*white circle*). (B) The bleb reached to fovea region, detached one side of the fovea and stopped (*white circle*). The *red circle* indicates a small BSS droplet (back flow) under PFCL during injection. (C) The bleb became smaller (*white circle*), and the BSS droplet increased the size once the cannula was taken out. (D–F) Intraoperative OCT images. *Green line* and *pink line* in D indicate the OCT images in E and F, respectively. *White triangles* in E and F indicated the mild intra retina edema. The *white arrow* in F indicates the small fovea tear.

**Figure 5.**
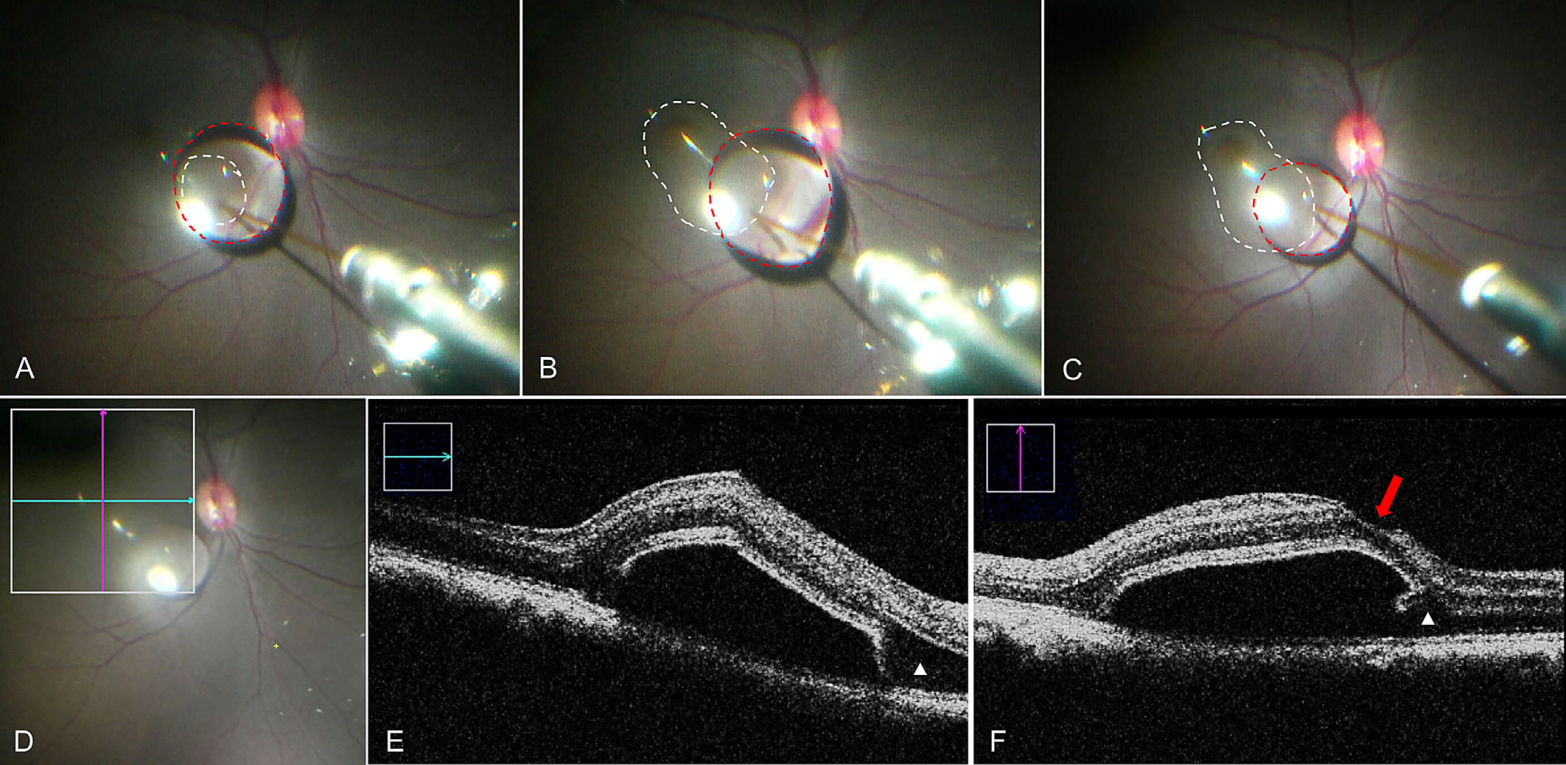
Foveal detachment raised under Condition 5. (A) The retinal bleb was initiated at superior nasal side (*white circle*). The red circle indicates a (accidentally spilled) BSS droplet under PFCL before injection. (B) The bleb reached to fovea region, detached one side of the fovea, and stopped (*white circle*). The *red circle* indicates that the BSS droplet became bigger during the injection because the back flow. (C) The bleb size did not change much (*white circle*), but the BSS droplet size became significant smaller after the cannula was removed. (D–F) Intraoperative OCT images. *Green line* and *pink line* in D indicate the OCT images in E and F, respectively. *White triangles* in E and F indicate the minimum intraretinal edema. The *white arrow* in F indicates that the fovea structure was intact and the ELM was well continued below the fovea.

Bleb retinal detachment (bRD) at the posterior pole was created by subretinal injection of ophthalmic grade balanced salt solution (BSS) using an extendible 38 G subretinal injection cannula (Cat. no. 3247; MedOne Surgical Inc., Sarasota, FL, USA) or 41 G dual bore cannula (Cat. no. 1701.01; Dutch Ophthalmic Research Center (D.O.R.C.), Zuidland, the Netherlands) under conditions specified in Table 1. The cannula was connected to a 1 mL MicroDose Kit (Cat. no. 3275; MedOne Surgical, Inc.) or 1 mL Tuberculin syringe (Terumo Syringe, Cat. no. SS+01T; Terumo Europe, Leuvin, Belgium) prefilled with BSS,^24^ or a gastight 0.1 mL Hamilton syringe (Cat. no. 80601; Hamilton Company, Inc., Reno, NV, USA),^29^ ensuring that all air bubbles have been evacuated from tubing. In both automated and manual injections, the cannula was inserted under the retina followed by the fluid injection. The bleb was gently raised, gradually controlled by foot pedal at 6 to 10 pounds per square inch (psi) from the vitrectomy machine (Constellation, Alcon) using the MicroDose Kit or manually injected by an assistant using either a Tuberculin or Hamilton syringe, to extend about 3 to 4 optic disc diameter (DD) (about 1+ quadrant of the posterior pole) to deliberately involve the fovea. Different conditions of vitreous tamponade (BSS, air, perfluoro-octane [Okta-Line; Bausch & Lomb]), as specified in Table 1, were used while bRDs were induced. The intraocular pressure (IOP) was set at the vitrectomy machine at different conditions range from 0 to 40 mmHg (Table 1). All surgical procedures were video recorded by a camera built in the surgical microscope. Retinal bleb formation, the separation of photoreceptors and the RPE, was confirmed by miOCT. Cube scans miOCT images (512 × 128 pixel, scan width 4 mm) were obtained at the fovea for detailed analysis (Table 2); miOCT images (crossed line scans) presented in Figures 1 to 5 are screenshots taken from live video mode. Preservative free triamcinolone (0.05 mL of 40 mg/mL) was injected intravitreally at the end of the surgery. A topical antibiotic ointment (Tobradex, Tobramycin and dexamethasone; Alcon) and 2% homatropine eye drops (Isopto, Alcon) were applied to the treated eyes twice a day for five days after surgery.

**Table 1.** Summary of a Variety of Conditions for Submacular Neurosensory Retinal Detachment

Condition	Injector	Syringe Type	Method of Injection	Psi	Injection Volume (µL)	Tamponade	IOP (mm Hg)	No. of NHP Eyes
1	38g MedOne cannula	1 mL MedOne MicroDose Kit	Foot pedal	6–10	200–300	BSS	10–40	*n* = 4
2	38g MedOne cannula	0.1 mL Hamilton	Manual	n/a	30–70	Air	5–10	*n* = 4
3	38g MedOne cannula	0.1 mL Hamilton	Manual	n/a	20–40	Partial PFCL	5–10	*n* = 3
4	38g MedOne cannula	0.1 mL Hamilton or 1 mL Tuberculine	Manual	n/a	15–25	Partial PFCL	0–4	*n* = 3
5	41g dual bore cannula	0.1 mL Hamilton	Manual	n/a	15–20	Partial PFCL	0–4	*n* = 2

**Table 2.** Observations in Individual Cases Under miOCT

Condition	Animal ID	Surgeon/Assistant	Extent of Foveal Detachment	Foveal Tear	Cystoid Macular Changes
1	Case 1 OD	GT/foot pedal	Full	Yes	Yes, moderate,
1	Case 2 OD	BS/foot pedal	Half	Yes	Yes, mild
1	Case 3 OD	GT/foot pedal	Nearly full	Yes	Yes, moderate
1	Case 4 OD	BS/foot pedal	Nearly full	Yes	Yes, moderate
2	Case 5 OD	BS/ZL	Nearly full	Yes	Yes, mild
2	Case 5 OS	GT/BS	Nearly full	Yes	Yes, moderate
2	Case 6 OD	GT/BS	Full	Yes	Yes, mild
2	Case 7 OD	BS/ZL	Full	Yes	Yes, severe
3	Case 8 OD	BS/GT	Full	Yes	Yes, severe
3	Case 9 OD	GT/BS	Full	Yes	Yes, moderate
3	Case 10 OD	BS/XS	Full	Yes	Yes, mild
4	Case 10 OS	GL/XS	Nearly full	No	Minimum
4	Case 11 OD	BS/XS	Nearly full	Yes	Yes, mild
4	Case 11 OS	GL/XS	Nearly full	No	Minimum
5	Case 12 OS	GL/himself	Nearly full	No	Minimum
5	Case 13 OS	GL/himself	Full	No	Minimum

### Postoperative OCT

Spectral domain OCT (SD-OCT) follow-up images at fovea were obtained using Spectralis (Heidelberg Engineering, Inc., Heidelberg, Germany) with a 30° lens. The proprietary follow-up function of this instrument was able to image SD-OCT scans at the same retinal location at multiple examine time points.

### Histopathological Examination

At one month after operation, 11 of 13 animals were sacrificed in deep anesthesia and perfused with 4% paraformaldehyde or 10% formalin, the eyes were enucleated, and entire globes were further fixed in the same fixative overnight. Full-thickness samples (retina→sclera) at fovea were cut and embedded in paraffin. Sections were cut at 5 µm with a microtome (Leica RM2255; Leica, Wetzlar, Germany) and further stained with hematoxylin and eosin.

## Results

Submacular neurosensory retinal detachment was induced with a variety of conditions as described in Table 1 and shown in Figures 1 to 5, as well as Supplementary Videos S1 to S5. Sixteen eyes of 13 cynomolgus macaques were available for the study based on availability of obtained miOCT data. Three different surgeons (BS, GT, GL) together with five different surgical assistants (GT, BS, GL, XS, ZL) were collectively involved in performing the maneuver, whereby no particular surgeon and assistant combination was found to be superior with regard foveal preservation when assessed by miOCT. All surgeons were following a previously established guideline,^11^ adapted in this study from human to macaque eye,^30^ to initiate submacular injection at least ≥3 mm away from the foveal center to avoid tangential retinal stretching.

In condition 1 (Table 1, Fig. 1, Video S1), with a foot pedal controlling the subretinal injection through the vitrectomy machine (Alcon Constellation) connected to the MicroDose Kit, the bleb developed rapidly in all eyes. The resulting subretinal fluid wave of the bRD initiated superotemporally about three to four DD away from the fovea then stopped immediately after passing underneath to the fovea in all eyes. Following built-up of additional subretinal fluid, a microtear of the fovea was noted in all eyes in the surgeon's view of the fundus, immediately followed by a shallowing of the bRD (Supplementary Video S1). Viscous fluid injection mode settings on 10 psi or subsequently 6 psi (the lowest possible) at the vitrectomy machine produced similar results. A foveal tear was thereafter confirmed on miOCT in all eyes under condition 1 (Fig. 1). It was noted that higher IOP settings at the vitrectomy machine (up to 40 mm Hg in condition 1) resulted in greater (vertical) bRD heights. Moreover, despite well-documented steadiness of the 38 G cannula tip in the retinotomy of each surgeon on video for condition 1 (GT and BS), the injected subretinal fluid volumes varied greatly (200–300 µL), suggesting some efflux of the subretinally injected BSS into the vitreous cavity.

With the aforementioned taken together, we hypothesized that reducing the IOP settings on the vitrectomy machine to 5 to 10 mm Hg and using a 10 times lower volume syringe (0.1 mL Hamilton) would reduce tangential retinal stretching, thus avoiding the foveal laceration. We further speculated that the use of a vitreous tamponade causing increased surface tension on the vitreoretinal interface might stabilize the neural retina and guide the subretinal injection into a more lamellar flow to facilitate the spread of the fluid wave beyond the fovea.

By performing a complete fluid-air exchange and meticulous removal of residual fluid trickling down to the posterior pole, this allowed the formation of a more controlled and gradual subretinal bleb in condition 2 (Table 1, Fig. 2, Supplementary Video S2). Of note in condition 2 was the significant reduction in subretinal injection volume (approximately 30 to 70 µL) for a bRD comparable to the volume in condition 1. However, cystoid macular changes were noted on miOCT (Fig. 2), and all eyes (*n* = 4) developed a foveal microtear.

Given the higher specific gravity of octaline (a perfluorocarbon liquid [PFCL] commonly used in vitreoretinal surgery)^31^ compared to saline solution, in condition 3 we hypothesized that the stabilizing effect of increased surface tension with air in condition 2 might be enhanced be an additional downward vector exerted by PFCL. The subretinal injection volume indeed decreased to 20 to 40 µL for a bRD size comparable to previous conditions. Similar to previous conditions, after passing underneath the fovea, the subretinal fluid wave would not extend beyond it; instead the bRD enlarged centrifugally (Supplementary Video S3). Attention had to be given to a strictly subretinal position of the canula tip during the injection, because accidental injection from an epiretinal location resulted in few instances in trapped subretinal PFCL droplets. However, epiretinal egress of fluid from the cannula tip would trap a BSS droplet at the vitreoretinal interface, underneath the PFCL (Supplementary Videos S4 or S5). Overall, the bleb formation was somewhat slower than previous conditions (compare Supplementary Videos S3 to S1 or S2), enabling an even more controlled subretinal injection. However, cystoid macular changes were still detectable in all three cases (Table 2, Fig. 3), albeit with a lower severity of foveal microtears.

In condition 4 (Fig. 4 and Table 1), the IOP was lowered further to less than 4 mm Hg. This reduced the surgeon-perceived manual force required to initiate the subretinal bleb detachment. Moreover, when the surgeon himself (GL)—without an assistant—manually performed the subretinal injection under chandelier illumination in two of four instances, there was greater perceived control over the injection maneuver. The subretinal injection volume in this condition further reduced to 15 to 25 µL, when measured using a 100 µL Hamilton syringe. Comparable to previous conditions, the fluid wave of the bRD stopped progressing right after foveal detachment (Supplementary Video S4). On assessment with the miOCT, the additional IOP lowering seemed to further reduce the extent and incidence of cystoid macular changes (graded as minimal to mild in all three cases) and foveal microtears (one case with a tiny microtear and two with confirmed intact foveal structure; Fig. 4 and Table 2).

To ensure subretinal injection under a constantly low IOP to avoid tangential retinal stretching (that we hypothesized to result in foveal trauma), condition 5 (Table 1, Fig. 5) used a 20/41 G dual-bore cannula to create the bRD. The dual-bore construction allows reflux of fluid from the vitreous cavity out of the eye in exchange for the BSS injected into the subretinal space, thus presumably avoiding an overt vertical increase in bleb size with increasing IOP. The retinal bleb was initiated at the disc, central of the temporal vascular arcade. As the bleb reached under the fovea, it stopped progressing further, similar to all previous conditions (Supplementary Video S5). On miOCT imaging, only minimal intraretinal cystic changes could be detected in both animals parafoveally (Table 2), where the fluid wave typically came to a stop (Figs. 5A–5C). The foveolar structure however was intact in both NHPs and the external limiting membrane (ELM) was not disrupted below the fovea on miOCT (Figs. 5D–5F). Starting another bRD with subretinal injection from the opposite end then allowed for complete detachment of the fovea without significant additional parafoveal intraretinal alterations.

**Figure 6.**
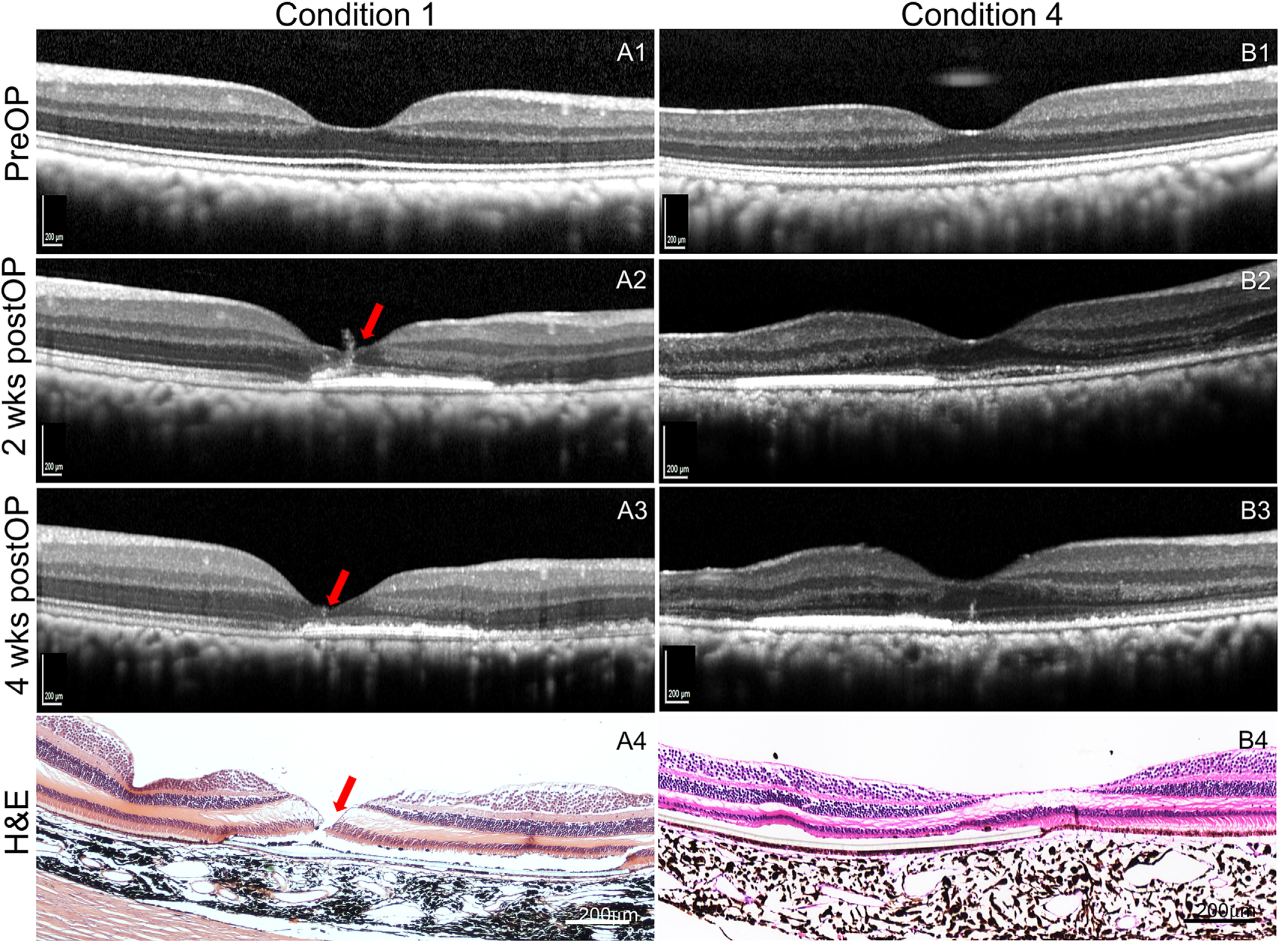
Postoperative follow-up on foveal structure with SD-OCT and histology. A1 to A4 and B1 to B4 are images of fovea structure on Condition 1 and 4, respectively. (A1–A3, B1–B3) SD-OCT images of the fovea at the same scanning position followed by tracking mode in Heidelberg Software. A4 and B4 are hematoxylin and eosin (H&E)–stained images at the fovea. *Red arrows* in A2 to A4 indicate the fovea lesion on OCT images and confirmed by H&E histology in Condition 1. Fovea structure was intact on OCT images and confirmed on histology in Condition 4 (B2–B4). Foveal dehiscence (A4) reappeared despite perfusion fixation for histology, the artifactual neurosensory detachment, and outer retinal cystoid edema in A4 and B4 are known processing artifacts. *Scale bar*: 200 µm.

To postoperatively evaluate the effect of above surgical conditions on foveal microstructure, repetitive SD-OCT and histology at four weeks were performed. The foveal microtear noted in condition 1 resulted in full-thickness disruption of foveal reflectivity at two weeks and significant outer nuclear layer thinning despite an almost continuous ELM on SD-OCT at four weeks. On paraffin-section histologic study, the foveal dehiscence reappeared despite perfusion fixation for histology, the artifactual neurosensory detachment and outer retinal cystoid edema are known processing artifacts. By contrast, in condition 4 where in two of three instances no foveal tear was noted during surgery, SD-OCT reflectance patterns on intraretinal foveal morphology at two and four weeks were almost comparable to preoperative images, or an unoperated eye (Supplementary Fig. S1). Likely because of residual subretinal fluid, a disruption of the subfoveal ellipsoid and interdigitation zone can be discerned at two weeks in the animal shown in (Fig. 6B2), which fully resolved at four weeks, both on SD-OCT and histology (Figs. 6B3, 6B4).

## Discussion

Integrated intraoperative optical coherence tomography (miOCT), allowed us to dynamically evaluate the microstructural changes in the macula during a subretinal procedure in NHP. It also allowed us real-time evaluation of various surgical maneuvers, thereby revealing structural changes that may no longer be present on postoperative OCT. This has been demonstrated in vitreomacular interface surgery such as epiretinal membrane, macular holes, and vitreomacular traction, where miOCT both identified additional membrane for peeling and prevented unnecessary surgical procedures once the surgical goal had been met.^32^^,^^33^ More complex retinal procedures such as retinal biopsy, retinal prosthesis placement, and delivery of subretinal therapeutic like gene therapy and stem cell therapy have also been described to be improved with miOCT feedback.^22^^,^^23^^,^^34^^,^^35^ The iOCT can provide live information on intraoperative anatomy and tissue/implant interface, confirming the depth, location, and amount of therapeutic product delivered. This enables the surgeon to make real-time decisions on surgical maneuverers that can avoid complication such as excessive surgical peeling, injection therapeutics at the wrong tissue level, and incorrect implant positioning and depth. In this study, we have evaluated subretinal injections in the NHP macular to optimize surgical techniques and reduce complications.

The fovea of healthy NHP appeared to be a very vulnerable structure during submacular injections of BSS, as demonstrated by miOCT imaging. With proper subretinal placement of the injection cannula before injection and an appropriately sized retinotomy, the subretinal fluid wave can reach and detach the fovea, after which it stops spreading further. At this stage, microstructural changes to the fovea are observed. Despite adjusting and thereby fine-tuning conditions for subretinal fluid injection, some degree of iatrogenic trauma was consistently demonstrated in this study with submacular BSS injections in 16 NHP eyes. Factors reducing the damage extent included lower syringe fluid volume (0.1 mL vs. 1 mL), reduced intraocular pressure (4 mm Hg vs. 10-40 mm Hg), initiating the bleb further away from the fovea and on the nasal side of the fovea (avoiding the papillomacular bundle), intravitreal tamponade agents and a dual bore subretinal injection cannula (Tables 1 and 2). We hypothesize that this submacular bleb spreading resistance at the parafovea is related to a biological variation in photoreceptor-RPE adhesions. Optimization of surgical instrumentation alone can only address the mechanical forces involved.

Surgically detaching the fovea has relevance to both retinal gene and cell therapy. Complete coverage of the macula with these Advanced Therapy Medicinal Products (ATMPs) in patients with better preserved vision will unavoidably need to involve fovea. The gene therapy field was first to recognize foveal vulnerability during RPE65 gene delivery in early clinical trials and suggested PFCL tamponading for protecting the macula,^12^ albeit without precise surgical technique disclosure. Ehlers et al.^23^ described miOCT-monitored subretinal injection of recombinant tissue plasminogen activator (rtPA) for AMD-related submacular hemorrhage in four cases without adverse surgical events, likely due to the already detached nature of the macula. Gregori et al.,^36^ however, demonstrated an impending macular hole/cystoid foveal changes with miOCT—similar to those observed in our series—in a patient undergoing gene therapy for choroideremia. Kashani et al.^37^ noted by using the miOCT in a series of 16 atrophic AMD patients during submacular placement of an embryonic stem cell–derived RPE patch subretinal adhesion spots (likely related to the underlying disease process) to prevent placement of the cell therapeutic construct.

Although these aforementioned clinical observations were in diseased tissue, surprisingly little systematic effort was undertaken to specifically optimize the rather traumatic effect of subretinal BSS injection in foveate preclinical animal models.^38^ Perhaps the best account toward that is the work by Ochakovski et al.^21^^,^^39^ who studied submacular injections in 18 cynomolgus macaques for gene therapy purposes, most of which included the fovea and found that subretinal injection for gene therapy does not cause clinically significant outer nuclear layer thinning. A careful analysis of their supplemental data however suggests that fovea-involving bleb retinal detachments were created from three individual bleb initiation sites (at the superior and inferior arcade, as well as temporal from the fovea) that reached confluence at the foveal center. Although miOCT monitoring was not described in that study, postoperative SD-OCT analysis revealing foveal disruption and thinning was classified as an outlier phenomenon. A detailed inspection of the ellipsoid zone reflectivity patterns may suggest that a skillfully created bleb detachment with vitrectomy machine controlled subretinal injection at 1 to 2 psi to just have reached the fovea, but the fluid wave has then not been allowed to extend further. In similar work in NHP by Takahashi et al., a minimal subretinal injection pressure of 6 psi was found necessary to create a small bleb retinal detachment through an inner limiting membrane peeled midperipheral retina (from epiretinal) without creating ellipsoid zone disruption.^27^ By contrast, Xue et al.^11^ described the use of 12 to 16 psi to detach dystrophic retina in choroideremia patients, whereas Davis et al.^40^ later mentioned that range to be 12 to 18 psi for RPE65 or choroderemia patients, and both of these authors reported detachment of the macular region observing foveal disruption on miOCT or SD-OCT in the human fovea.^11^ In our experience, using the Alcon Constellation machine viscous fluid injection setting at 6 to 10 psi resulted in consistent foveal tearing in NHPs. This suggests that foveal compliance and tolerance of tangential stretching may differ significantly (perhaps by three times or more) between human and macaques, possibly also for healthy versus diseased tissue.^27^^,^^39^

Another observation we had was that bleb height was higher (relative to its area) with higher IOP settings at the vitrectomy machine. In other words, at lower IOP settings, retinal blebs tended to have a shallower configuration. A typical intravitreal injection in humans with 0.05 mL can double the IOP^41^; however, volumes beyond this often require a paracentesis. An increasing IOP during the subretinal injection may require greater force to detached the retina and result in more turbulent flow through the 38 G needle. Dropping the IOP to zero and the use of octaline as a heavy liquid vitreous tamponade would reduce these forces, but it did not entirely abolish the iatrogenic damage at the parafoveal outer retinal edge. Xue et al.^11^ were the first to describe the use of a dual bore cannula for subretinal gene therapeutic delivery to eliminate viral particles escaped into the vitreous cavity as a result of high IOP during the procedure. Its construction allows the simultaneous influx and egress of identical fluid volumes. This subtle adjustment then contributed to the least traumatic foveal elevation observed by miOCT in our series. With the above taken together, reducing subretinal injection flow volume through controlled manual injection with a 100 µL syringe, PFC tamponading, and a stable low IOP jointly reduced the iatrogenic foveal trauma on miOCT.

A peculiar, yet standard observation was the resistance of the fovea to detach fully with a single fluid wave. The miOCT would always demonstrate residual parafoveal outer retinal adhesions to the RPE along with varying degrees of intraretinal trauma. We speculate that as the bleb detachment reaches the transition of the foveal wall to the parafoveal region, the force required to detach the neurosensory retina exceeds the horizontal intercellular adhesion forces of the Müller cells (MC) at the fovea externa, resulting in various degrees of trauma ranging from cystoid edema to foveal microtears.^42^ The OCT lesion patterns observed throughout our subretinal injection conditions in fact appear to resemble in some aspects those of macular hole formation,^43^ suggesting that distribution of specialized MC populations and their respective mechanical strength may play a role in foveal vulnerability during subretinal injection.

Although the mechanical forces at play are likely not sharing the same vectors, tears in the ELM seem involved in both instances. Xue et al.^11^ described transient macular thickening in choroideremia patients in the initial days after the subretinal gene transfer procedure, albeit without apparent subsequent clinical relevance. The rarely reported foveal microtears from human gene therapy trials suggest that the human macula may be less susceptible to trauma than in NHPs, which is a caveat for applying our findings in NHPs for subretinal injections performed in humans. However, modifying subretinal injection techniques to minimize foveal trauma will still be desirable.

We hypothesize that the photoreceptor-RPE adhesion in the parafoveal zone is much stronger than the rest of the retina, and this is consistent with the observation that patients with vitreomacular traction (VMT) on full-thickness macular holes (MH) will typically not proceed to retinal detachment, in contrast to peripheral retinal tears. Moreover, submacular injection of BSS in large and or persistent macular holes seems to facilitate their closure by releasing parafoveal subretinal adhesions.^44^ By contrast, successful release of VMT from MH by Ocriplasmin intravitreal injection is occasionally associated with a transient increase in the MH base diameter due to its additional action on the interphotoreceptor matrix (IPM).^45^^,^^46^ All of these observations may, in addition to the above-discussed MC role, point to a variation in the composition of the parafoveal IPM.

This study is limited by many variables evaluated simultaneously and numbers of NHPs available to assess each condition. The “multiple surgeons factor” could also have influenced the outcome, albeit surgery was performed in an otherwise consistent manner, by surgeons who all had adequate surgical experience. A surgeon-controlled versus assistant-performed subretinal injection requires further study, because dynamic fluid kinetics matter particularly in the early phase of bRD formation. Last, although the macular anatomy in NHP is very similar to human, subtle interspecies differences may nevertheless limit the applicability of our work to human surgery.

In conclusion, this study demonstrated the real-time changes in the macula during subretinal fluid injection, which results in foveal trauma in NHP with conventional technique derived from human clinical trials. A slower, more controlled bleb retinal detachment was achieved initiating the injection further away from the fovea with a dual bore cannula, which ensured reduced fluid flow under a steady low IOP and PFCL tamponade, thus substantially reducing the amount of traumatic changes as evidenced on miOCT. Adopting these modifications to subretinal injection technique in human clinical trials could potentially reduce macular damage and improve the therapeutic outcomes of subretinal injection procedures by, for example, enabling coverage of a larger retinal area with identical ATMP volume.

## Supplementary Material

Supplement 1

Supplement 2

Supplement 3

Supplement 4

Supplement 5

Supplement 6
