# Supplementary material for: Hints for Gentle Submacular Injection in Non-Human Primates Based on Intraoperative OCT Guidance
Source: Transl Vis Sci Technol. 2021 Jan 7;10(1):10. doi: 10.1167/tvst.10.1.10 (PMC7804573; doi:10.1167/tvst.10.1.10)
Supplement: Supplement 1 [file tvst-10-1-10_s001.pdf]

Supplementary material

Supplementary figure

Figure S1

Normal NHP foveal structure in SD-OCT (A) and H&E histology (B). Inset in (A) shows IR SLO image with green line showing location of vertical line scan. The corresponding histology is also a vertical section. Note that SD-OCT and histology are not to scale, given OCT image processing (compare vertical and horizontal scale bar). Retinoschisis-like macular edema on histology is a post-mortem artefact.

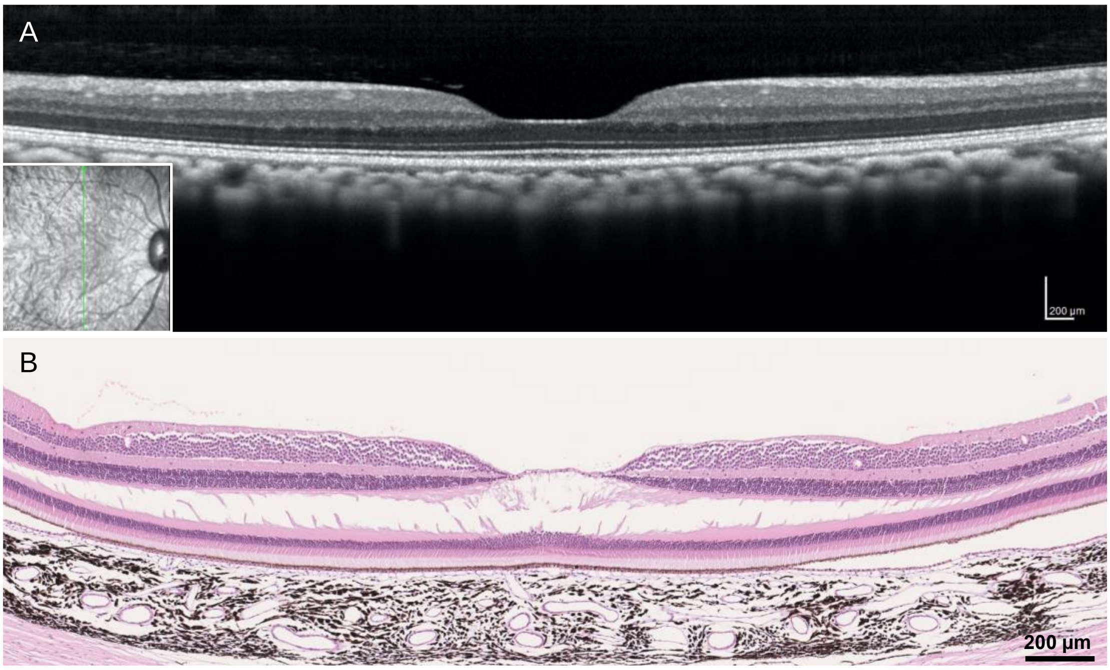

Supplementary videos

Video S1

Condition 1 (Table 1), foot pedal vitrectomy machine-controlled submacular BSS injection in a right eye.

The injection is initiated superotemporal, 2-3 DD from the macula. Note, as the fluid wave reaches under the fovea it stops and the bRD height builds up to the point when the fovea tears. Thereafter an immediate shallowing of the bRD is observed, but no further extension of the bleb.

Video S2

Condition 2 (Table 1), manual submacular BSS injection with 0.1ml syringe under air tamponade in a right eye.

A second injection is initiated superotemporal, 2-3 DD from the macula, since the first (already visible) bleb initiated 3-4 DD inferonasally from the fovea stopped progressing once it reached under the fovea. Again note, as the fluid wave reaches the fovea it connects with the inferonasal bRD, but resists to detach a parafoveal adhesion supertemporally. Note that air tamponade adversely affects intraoperative visualization of the posterior pole.

Video S3

Condition 3 (Table 1), manual submacular BSS injection with 0.1ml syringe under octaline (PFCL) tamponade in a right eye.

The injection initiated peripapillary at the inferior temporal vascular arcade ca. 4mm from the fovea stopped progressing once it reached under the fovea.

Instead, as bleb height builds up the fluid wave extends centrifugal. Note that no BSS reflux at the retinotomy or fluid egressing through the foveal roof underneath the octaline can be observed.

Video S4

Condition 4 (Table 1), manual submacular BSS injection with 0.1 ml or 1 ml syringe under octaline (PFCL) tamponade in a right eye.

The injection initiated peripapillary at the superior temporal vascular arcade ca. 4 mm from the fovea stopped progressing once it reached under the fovea. At this point the injection is deliberately stopped to avoid disruption of foveal structure. As the cannula is retracted from the retinotomy, a small BSS droplet becomes lodged at the PFCL/retina interface.

Video S5

Condition 5 (Table 1), manual submacular BSS injection with a dual bore cannula on a 0.1 ml syringe under octaline (PFCL) tamponade in a left eye.

As the cannula approaches the retinal surface, a small BSS droplet becomes lodged at the PFCL/retina interface. The injection is then initiated peripapillary at the superior temporal vascular arcade ca. 4 mm from the fovea, but stops progressing once it reaches under the fovea. At this point the injection is deliberately stopped to avoid disruption of foveal structure. Note the directed spread, along with a relatively small bleb size and height, when compared to other conditions.
